# An Improved Diabatization Scheme for Computing the Electronic Circular Dichroism of Proteins

**DOI:** 10.1021/acs.jpcb.4c02582

**Published:** 2024-07-22

**Authors:** David M. Rogers, Hainam Do, Jonathan D. Hirst

**Affiliations:** † School of Chemistry, University of Nottingham, University Park, Nottingham NG7 2RD, United Kingdom; ‡ Department of Chemical and Environmental Engineering and Key Laboratory of Carbonaceous Waste Processing and Process Intensification Research of Zhejiang Province, University of Nottingham Ningbo China, Ningbo 315100, China; § New Materials Institute, University of Nottingham Ningbo China, Ningbo 315042, China

## Abstract

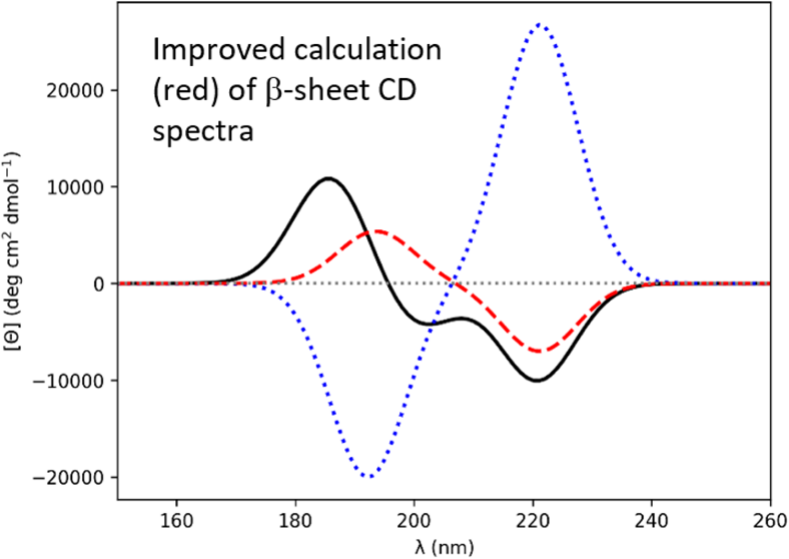

We advance the quality of first-principles calculations of protein electronic circular dichroism (CD) through an amelioration of a key deficiency of a previous procedure that involved diabatization of electronic states on the amide chromophore (to obtain interamide couplings) in a β-strand conformation of a diamide. This yields substantially improved calculated far-ultraviolet (far-UV) electronic circular dichroism (CD) spectra for β-sheet conformations. The interamide couplings from the diabatization procedure for 13 secondary structural elements (13 diamide structures) are applied to compute the CD spectra for seven example proteins: myoglobin (α helix), jacalin (β strand), concanavalin A (β type I), elastase (β type II), papain (α + β), 3_10_-helix bundle (3_10_-helix) and snow flea antifreeze protein (polyproline). In all cases, except concanavalin A and papain, the CD spectra computed using the interamide couplings from the diabatization procedure yield improved agreement with experiment with respect to previous first-principles calculations.

## Introduction

Electronic circular dichroism (CD) spectroscopy finds widespread utility in the biophysical characterization of protein structure,^1^ including systems as challenging as intrinsically disordered proteins.^2^ Statistics-based techniques can compute secondary structure content from the experimental CD spectrum of a biomolecule.^3−6^ For example, Zavrtanik et al.^7^ have recently developed a framework to improve the analysis of the helical content of alanine-rich peptides using a CD spectrum as input. Empirical-based techniques have been developed to predict protein CD spectra from protein atomic coordinates, for example, the SESCA method of Nagy and co-workers^8^ which can be compared to the PDB2CD method of Mavridis and Janes,^9^ and the recent KCD method of Jacinto-Méndez et al.^10^ Calculations of the CD spectra of polypeptides from first-principles^11^ afford a direct connection between molecular simulations and experiment, and thereby insights with a spatial and temporal resolution that could not be realized by experiment alone. Examples range from the photoisomerization of azo-peptides^12^ through to the binding of proteins to silica.^13^

Physics-based techniques have been developed to compute the infrared absorption and the vibrational CD of biomolecules.^14−17^ These approaches use results from quantum chemical calculations on small model peptides (quantum-mechanically computed force fields and atomic polar and axial tensors) to construct a formalism to apply to the study of larger biomolecules, including solvation effects. However, the techniques appear to be limited to application to study short peptides and segments of distinct secondary structure extracted from larger proteins. A framework based on exciton theory, outlined below, does not face such limitations.

Starting from an atomistic structure, which may come from experimentally determined coordinates from the Protein Data Bank (PDB) or from a molecular simulation, one can calculate the CD spectrum of a protein using an exciton framework.^18−20^ This has been implemented in the open-source DichroCalc software^21^ and a web-interface allows users to upload a protein structure or an ensemble of structures and compute the CD spectrum. In the exciton framework as it is applied to protein CD in the far-ultraviolet (far-UV), one considers the *n*π* (at 220 nm) and π_nb_π* (at 190 nm) electronic transitions on each peptide chromophore. These arise, respectively, from excitation from a lone pair on the carbonyl oxygen atom to the antibonding π* orbital and from excitation from the nonbonding π orbital of the amide group to the π*orbital. The transitions are used to construct an effective Hamiltonian. A key aspect is the calculation of the interaction energies between transitions; these couplings constitute the off-diagonal elements of the Hamiltonian matrix, with the diagonal elements comprising the electronic transition energies of the isolated chromophores.

Higher-energy singlet transitions on the amide chromophore have been included in the exciton framework to compute protein CD.^22^ The coupling of the *n*′π* (second lone pair on oxygen to π* at 123 nm) and π_b_π* (π bonding to π* at 129 nm) electronic transitions with the *n*π* and π_nb_π* electronic transitions did not improve the correlation with experimental spectra at wavelengths of 190, 208, and 220 nm for a set of 47 proteins. Charge-transfer transitions between two neighboring amide groups in a diamide have been considered for 10 secondary structures to yield transition parameters for exciton theory calculations to compute CD.^23^ For a set of 31 proteins, where synchrotron radiation CD (SRCD) spectra were available, the addition of the charge-transfer chromophores to the exciton framework made little difference to the correlation of computed with experimental spectra for wavelengths above 190 nm. The agreement between the computed and the experimental spectra improved between 170 and 190 nm, where at 175 nm the Spearman rank correlation coefficient is 0.44 without charge-transfer and 0.80 with charge-transfer chromophores incorporated.

Kumar and co-workers have performed two studies^24,25^ using time-dependent density functional theory (TDDFT) to compute the CD for cationic tripeptides in β-strand and polyproline conformations, taking the effect of solvent (water) into account. They concluded that additional electronic transitions, to the valence (*n*π* and π_nb_π*) amide transitions, and explicit solvent molecules, that contribute to the hole/particle character of the ground and electronically excited states of the amides, are required to accurately compute the CD for the cationic tripeptides when in a β-strand or a polyproline conformation. As mentioned above for the work of Keiderling and co-workers,^14−17^ the size of the studied system is limited to a short (tri)peptide (plus solvent) and not a whole protein. The role of solvent in the electronic transitions was interpreted from a natural transition orbital (NTO) analysis of the one-body transition density matrices. An alternative orbital localization procedure (such as Boys or Pipek-Mezey), may lead to the interpretation that the orbitals, once transformed, are more localized. Comparison of Kumar and co-workers’ results with a sophisticated single-reference wave function method, such as DLPNO-STEOM-CCSD that can be applied to study the excitation spectra of large molecules, would be interesting and help elucidate the role of water in the electronic transitions for β-strand and polyproline conformations. In the exciton framework presented herein, we build on previous studies employing two amide transitions (*n*π* and π_nb_π*) to improve the computation of protein CD and to establish the limits of the approximations within the exciton framework and to assess the origin of those limitations.

To improve the description of nearest neighbor interactions between two electronic transitions localized on each individual peptide bond, we recently applied a diabatization scheme to a full *ab initio* calculation on a diamide model.^26^ While an encouraging improvement over the standard DichroCalc parameter set was achieved for α helical conformations, the CD spectra of β-strand conformations computed using the diabatic interamide interactions were qualitatively incorrect. Diabatization in our case involves transformation of the eigenstates of the diamide to a diabatic basis. The approach can treat multiple excited states and it also takes into account both short- and long-range effects of the excitonic couplings. There are several strategies that can be employed to define the adiabatic-to-diabatic transformation. However, establishing a consistent set of phases is a general technical challenge in diabatization schemes, which is well recognized.^27,28^ Consequentially, the signs of the excitonic coupling from the diabatization are not well-defined.

**Figure 1:**
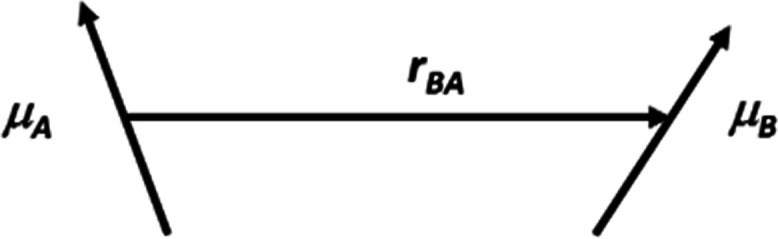
Two electric transition dipole moment vectors, **μ**_**A**_ and **μ**_**B**_, separated by a vector ***r***_**AB**_.

In CD, the rotational strength, *R*, is directly related to the intensity of a band in the spectrum. It is analogous to the dipole strength in electronic absorption spectroscopy. For two nondegenerate chromophores, A and B, with rotational strengths, *R*_A_ and *R*_B_, respectively
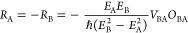
1where *E*_A_ and *E*_B_ are the energies of the transitions on chromophores A and B, *V*_BA_ is the associated interaction energy (which can be computed using the dipole–dipole approximation) and *O*_BA_ is called the optical factor. The optical factor was introduced by Schellman^29^ and has been helpful, for example, in theoretical considerations of the induced CD of DNA intercalators.^30^ It is the scalar triple product of the vector, ***r***_**BA**_, separating two chromophoric groups and the electric transition dipole moment vectors, **μ**_**A**_ and **μ**_**B**_, of the electronic transitions on each chromophore (Figure 1)
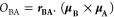
2Thus, the optical factor depends on the relative geometric arrangement of the two electric transition dipole moments. For example, if they are parallel or antiparallel, the optical factor is zero.

In this study, we propose that a difference between the *ab initio* (monomer) and the adiabatic (dimer) in orientation of the (**μ**_**B**_ × **μ**_**A**_) cross product indicates that an inconsistent phase has arisen from the diabatization process, which should be remedied by changing the sign of one (or other) of the electric transition dipole moments. Our aims are, first, to correct the discrepancy found in the CD spectrum for a β-strand structure computed using interamide couplings from the diabatization scheme presented previously,^26^ and, second, to apply the interamide couplings from the diabatization scheme to compute CD spectra for proteins whose experimental CD spectra are known. The latter is to assess the accuracy of the diabatization methodology compared to CD spectra computed using standard DichroCalc *ab initio* parameters.

## Methods

The electronic excited states of a set of conformations of the linear diamide *N*-acetylglycine *N*′-methylamide (NAGMA) have been previously calculated *ab initio* at the complete active space self-consistent field (CASSCF) level.^23,31^ These calculations, and particularly the associated electric transition dipole moments, provide the starting point for the diabatization procedure. The CASSCF calculations employed an atomic natural orbital (ANO) basis set contracted to 4s3p1d for C, N and O atoms, and to 2s1p for H atoms. Vertical transition energies were computed by multireference second-order perturbation theory (CASPT2) using state-averaged CASSCF reference wave functions. The state-averaged CASSCF wave functions described the ground state, the valence electronically excited states and the charge-transfer excited states of NAGMA. State-averaged CASSCF calculations accurately describe, for a given root or electronic state, any multireference character of the electrons and orbitals included in the active space, and include the effects of full configuration interaction within the active space. This CASSCF/CASPT2 approach has proven effective to describe the electronic transitions in the amide chromophore,^32,33^ in a diamide^31,34−36^ and in aromatic side chains of amino acids.^37^ All relevant parameters^23,26,31^ are available from the Dichrocalc Web site.^21^ The diamide conformations, which cover the major elements of protein secondary structure, are summarized in Table 1.

**Table 1:** Diamide Conformations for which *ab initio* Calculations of Their Excited States are Used in the Diabatization Process^a^

diamide	dihedral angles (ϕ/°, ψ/°)	conformation
**2a**	(180, 180)	fully extended
**2d**	(−135, 135)	β-sheet
**2e**	(−120, 120)	β-sheet
**2i**	(−74, −4)	3_10_-helix
**2j**	(−48, −57)	Pauling–Corey–Branson α-helix
**2k**	(−60, −60)	idealized α-helix
**2l**	(−62, −41)	Barlow–Thornton α-helix
**2m**	(−75, 145)	PPII helix

a Only the pertinent conformations, out of 13 in total, **2a** to **2m**, are shown. The Barlow–Thornton conformation has the average dihedral angles of helices in the PDB.^38^

We briefly recapitulate the diabatization process, which has been presented previously.^26^ It follows the work of Aragó and Troisi.^39^ A diabatic Hamiltonian, **H**^*D*^, is computed using a transformation of the adiabatic Hamiltonian, **H**^*A*^:
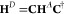
3

The unitary transformation matrix **C** relates the diabatic states (on the dimer) to the adiabatic states (on the dimer) and is computed by minimizing the difference between the adiabatic transition dipole moments on the dimer and the isolated monomer in the coordinate frame of the dimer.^39^ The adiabatic Hamiltonian is diagonal; the diagonal elements are the transition energies of the excited states in the dimeric system. The diabatization procedure gives the diabatic Hamiltonian with transition energies on the diagonal and the coupling between excited states in the diabatic representation in the off-diagonal matrix elements.

We assume that the cross product and optical factor for the dimer (adiabatic and from *ab initio* calculations on the diamide geometries) are correct for a dimeric system. The angle (phase) difference between monomer–dimer cross products may be due to the arbitrary nature of wave function phases in the monomer and dimer calculations. This would affect their interactions, and the diabatization, when considering the electronic coupling of amide 1 with amide 2. The dimeric *ab initio* calculations are assumed to have the correct behavior of the translation (dipolar, π_nb_π*) and rotation (quadrupolar, *n*π*) of transitioning electrons that contribute to the electric transition dipole moments that are important in determining rotational strength.

The dot product and/or the angle between the monomer and dimer (**μ**_***j***_ × **μ**_***i***_*)* cross products, where *i* and *j* refer to transitions, can be used to inspect their relative phase or orientation. An angle of ∼180° would indicate an inconsistency in the phases, which could be resolved by multiplying the monomer **μ**_***j***_ (and **μ**_***i***_) electric transition dipole moment components by −1 to change its sign. The electric transition dipole moment vectors **μ**_***j***_ and **μ**_***i***_ are taken from the DichroCalc parameter sets for the adiabatic NAGMA geometries, and from DichroCalc output for the two monomers in the coordinate frame of the dimer; the monomer electric transition dipole moments are taken from NMA4FIT2 parameters.^20^ The NMA4FIT2 parameters were derived from *ab initio* calculations on *N*-methylacetamide. Hereon we use “monomer” to refer to these. The amide oxygen atom for each amide is used as the reference point for the transition dipole moment vectors and is used to compute their separation, ***r***_***ji***_, where ***r***_***ji***_ = ***r***_***j***_ – ***r***_***i***_. Below, we consider the consequences of the diabatic couplings replacing the default DichroCalc Coulombic couplings computed using charges from the NMA4FIT2 parameter set, and where the diagonal transition energies are identical for each monomer.

The computation of CD spectra for the Ala_20_ model peptides employed the methodology as described by Rogers et al.^26^ The approach used for the example proteins is described below. The 13 sets of diabatic couplings replace the DichroCalc (*ab initio*) couplings for all nearest neighbor interamide interactions in the Hamiltonian. The Euclidean distance (on the Ramachandran plot) between protein (ϕ, ψ) and diamide (ϕ, ψ) was used to determine the closest diamide (ϕ, ψ) to the protein (ϕ, ψ), with the interamide couplings in the protein Hamiltonian being modified to the closest (ϕ, ψ) in the 13 diamide diabatic coupling sets.

The determination of the closest diamide (ϕ, ψ) proceeds as follows. For *N* residues (amino acids) there are *M* = *N* – 1 peptide bonds. Interaction of peptide bonds 1 and 2 is defined by (ϕ, ψ) of amino acid 2; interaction of peptide bonds (*M* – 1) and *M* by (ϕ, ψ) of amino acid *N* – 1. For the interaction between peptide bonds in DichroCalc, the first N-terminus (ϕ, ψ) (where ϕ is not defined) and all C-terminus (ϕ, ψ) (where ψ is not defined) are neglected, the latter as a consequence of chain borders. The Euclidean distance between protein (ϕ, ψ) and diamide (ϕ, ψ) is used to find the closest diamide (ϕ, ψ).

The DichroCalc Hamiltonian has dimension 2*N* – 2 × 2*N* – 2 for *N* amino acids. The DichroCalc Hamiltonian off-diagonal elements [*i, j*] (upper triangle) for peptide bond *M* coupling with peptide bond *M* + 1 are [(2*M*) – 1, (2*M*) + 1], [(2*M*) – 1, (2*M*) + 2], [2*M*, (2*M*) + 1] and [2*M*, (2*M*) + 2] for, respectively, the *n*π_1_^*^-*n*π_2_^*^, *n*π_1_^*^-π_nb_π_2_^*^, π_nb_π_1_^*^-*n*π_2_^*^ and π_nb_π_1_^*^-π_nb_π_2_^*^ couplings. These Hamiltonian matrix elements are replaced by the diamide diabatic couplings (as are the lower triangle of the Hamiltonian) to compute the CD. Table S1 displays a segment from the DichroCalc Hamiltonian matrix for myoglobin before and after modification.

## Results

Table 2 shows the optical factors, i.e., the scalar triple product, ***r***_***ji***_. (**μ**_***j***_ × **μ**_***i***_), and dipole–dipole coupling interactions, *V*_dd_, for the fully extended diamide **2a** calculated with the expression shown in eq 4 using in one case the electric transition dipole moments from the monomer and in the other case those from the NAGMA diamide.
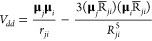
4

**Table 2:** Optical Factor and Dipole–Dipole Interaction for the Inter-amide Couplings for the Fully Extended Diamide **2a**, Computed Using the Monomer and Dimer Electric Transition Dipole Moments

	quantity	*n*π_1_^*^-nπ_2_^*^	*n*π_1_^*^-π_nb_π_2_^*^	π_nb_π_1_^*^-*n*π_2_^*^	π_nb_π_1_^*^-π_nb_π_2_^*^
monomer	optical factor/Debye^2^ Å	0.001	3.1	0.07	0.006
	coupling/cm^–1^	–2.3	0.1	0.1	–103.8
dimer	optical factor/Debye^2^ Å	0.002	–2.8	–0.7	0.03
	coupling/cm^–1^	–1.2	–0.2	1.0	499.8

The cross products used to calculate the optical factors are provided in the Supporting Information (Table S2) along with the locations of the reference oxygen atoms (Table S3) used to compute the vectors between neighboring amide chromophores. For diamide **2a** (fully extended: ϕ = 180°, ψ = 180°), the optical factor for the *n*π_1_^*^-π_nb_π_2_^*^ coupling is the largest of the four couplings, because the electric transition dipole moments of the π_nb_π* transitions on the two chromophores are antiparallel, as are those of the two *n*π* transitions.

Over the 13 diamide conformations, there are often sign changes between optical factors computed using the monomer electric transition dipole moments and those from the NAGMA dimer, which may be related to the difference in angle between the dipole–dipole cross products arising from the two regimes. In four cases, this difference in angle is greater than 170°. Of particular interest are diamides **2d** (−135°, 135°) and **2l** (−48°, −57°), corresponding, respectively, to the β-strand and Pauling–Corey–Branson α-helical conformations. Table 3 shows, for these two cases, the optical factors and the dipole–dipole coupling interactions computed using the monomer electric transition dipole moments and those from the NAGMA dimer. In addition, the table shows the difference in angle between the dipole–dipole cross products.

**Table 3:** Optical Factor and Dipole–dipole Interaction for the Inter-amide Couplings for Diamides **2d** (−135°, 135°) β-Strand and **2l** (−62°, – 41°) α-Helix Computed Using the Monomer and Dimer Electric Transition Dipole Moments^a^

	quantity	*n*π_1_^*^-nπ_2_^*^	*n*π_1_^*^-π_nb_π_2_^*^	π_nb_π_1_^*^-*n*π_2_^*^	π_nb_ π_1_^*^-π_nb_π_2_^*^
2d (−135°, 135°) β-strand					
monomer	optical factor/Debye^2^ Å	–0.1	2.5	–0.4	6.9
	coupling/cm^–1^	–2.0	–17.7	17.4	16.8
dimer	optical factor/Debye^2^ Å	–0.1	–2.4	–1.4	–6.9
	coupling/cm^–1^	–1.6	18.5	17.0	393.7
angle/°		17.1	146.5	19.0	175.8
2l (−62°, – 41°) α-helix					
monomer	optical factor/Debye^2^ Å	–0.1	–0.8	–0.0002	–23.6
	coupling/cm^–1^	1.8	–74.6	–9.9	519.6
dimer	optical factor/Debye^2^ Å	0.03	1.4	–2.2	–32.3
	coupling/cm^–1^	–1.6	–6.0	–3.5	–701.7
angle/°		171.2	118.5	118.0	16.2

a The angle between the dipole–dipole cross products is also shown.

**Figure 2:**
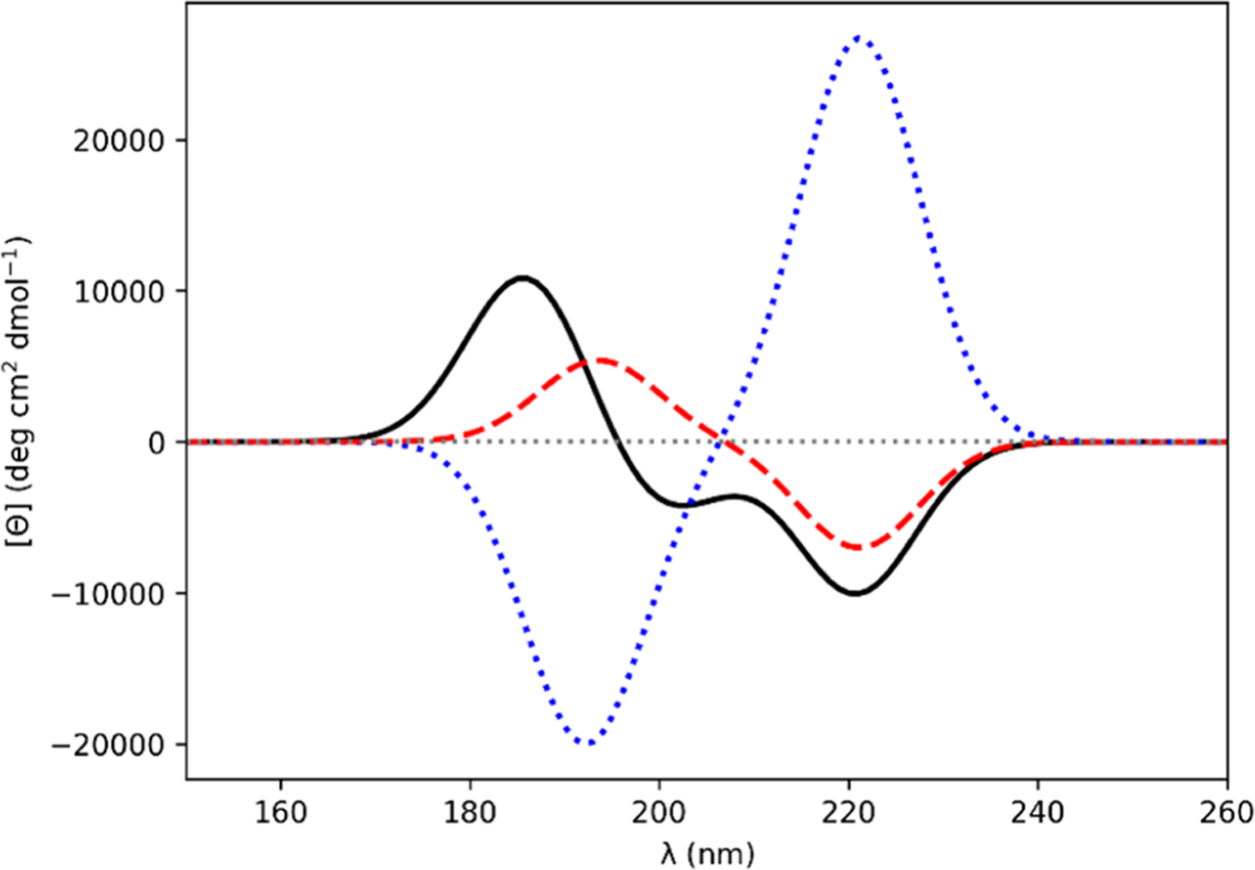
Computed CD spectra for β-strand Ala_20_ (−135°, 135°). Gaussians of fwhm of 9.0 nm were fitted to the rotational strength line spectra to obtain the CD spectra. *Ab initio* NMA4FIT2 (solid black line), “standard” diabatic (dotted blue line), diabatic with modified π_nb_π_2_^*^ electric transition dipole moment (dashed red line).

The dipole–dipole cross products from the monomer and dimer electric transition dipole moments relating to the π_nb_π_1_^*^-π_nb_π_2_^*^ interaction in **2d** (β-strand: −135°, 135°) differ in angle by 176°. This could be diagnostic of an inconsistency in phases. Thus, following our proposed prescription, we changed the sign of the monomer π_nb_π_2_^*^ electric transition dipole moment before the diabatization procedure. The resulting interamide coupling interactions from the diabatization and the CD spectra, computed using the couplings from diabatization, for Ala_20_, with all main chain dihedral angles set to (−135°, 135°), are shown in Table 4 and Figure 2, respectively. We have also explored the consequences of changing the sign of the monomer π_nb_π_1_^*^ electric transition dipole moment before the diabatization procedure, and the consequence of changing the sign of both moments. The coupling interactions that arise are also shown in Table 4.

**Table 4:** Inter-amide Interactions (cm^–1^) for Diamide **2d** (−135°, 135°) β-Strand^a^

method	*nπ*_1_^*^-*n*π_2_^*^	*n*π_1_^*^-π_nb_π_2_^*^	π_nb_π_1_^*^-nπ_2_^*^	π_nb_π_1_^*^-π_nb_π_2_^*^
Ab initio	72	–20	292	–907
diabatic	109	–279	–244	–89
ππ_2_^*^	109	279	–244	89
ππ_1_^*^	109	–279	244	89
ππ_2_^*^ and ππ_1_^*^	109	279	244	–89

a The first and second rows are from the DichroCalc *ab initio* and the standard diabatic approaches (as per Rogers et al.^26^), respectively. In the rows that follow, for the state(s) indicated, the monomer electric transition dipole moment has undergone a sign change before diabatization.

For the β-strand Ala_20_ structure corresponding to **2d**, the standard diabatic spectrum (Figure 2) has an intense negative band at 195 nm and an intense positive band at 222 nm, which disagrees qualitatively with experimental spectra for β-sheets. Changing the sign of the electric transition dipole moment of the π_nb_π_2_^*^ monomer state results in a CD spectrum with a positive band at 195 nm and a negative band at 222 nm (Figure 2) that more closely resembles a β-strand spectrum. Changing the sign of the electric transition dipole moment of the π_nb_π_1_^*^ monomer state gives a CD spectrum (data not shown) similar to that arising from the standard (i.e., unmodified) diabatic approach, but with less intense negative and positive bands. Changing the sign of both states gives a CD spectrum that appears to be the inverse of the standard diabatic spectrum.

**Figure 3:**
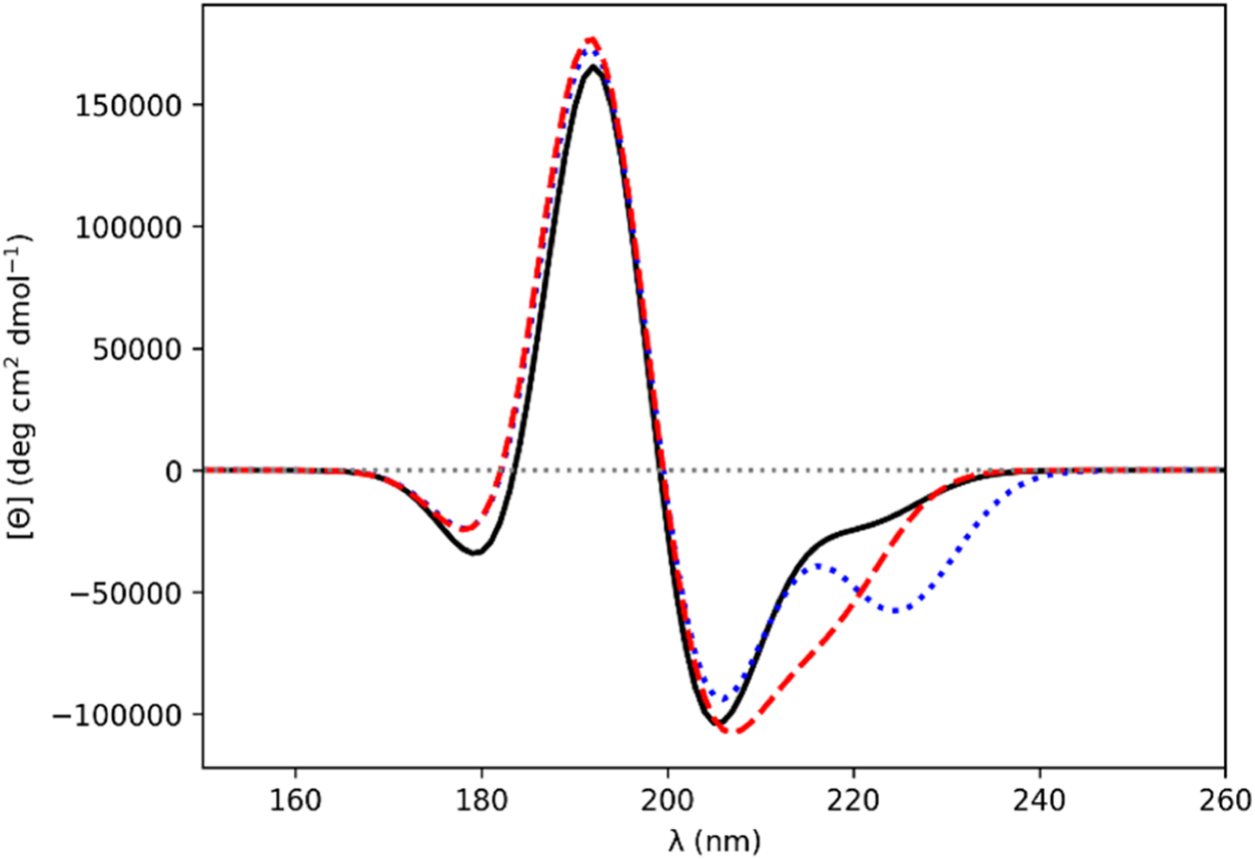
Computed CD spectra for Barlow–Thornton α-helix Ala_20_ (−62°, −41°). Gaussians of fwhm of 9.0 nm were fitted to the rotational strength line spectra to obtain the CD spectra. *Ab initio* NMA4FIT2 (solid black line), “standard” diabatic (dotted blue line), diabatic with modified *n*π_2_^*^ electric transition dipole moment (dashed red line).

Diamide **2l** has dihedral angles (−62°, −41°), corresponding to the Barlow–Thornton helix.^38^ The standard diabatic spectrum (Figure 3) features the double minimum, which is characteristic of an α-helix, at 205 and 225 nm. The dipole–dipole cross products from the monomer and dimer electric transition dipole moments relating to the *n*π_1_^*^-*n*π_2_^*^ interaction in **2l** differ in angle by 171°. In contrast to the strongly electric transition dipole allowed π_nb_π* transition, the *n*π* transition has a very weak electric transition dipole moment. Following our proposed prescription again, we changed the sign of the monomer *n*π_2_^*^ electric transition dipole moment before the diabatization procedure. The coupling interactions resulting from the diabatization and the CD spectra, computed using the couplings from diabatization, for Ala_20_, with all main chain dihedral angles set to (−62°, 41°), are shown, respectively, in Table 5 and Figure 3. Changing the sign of the electric transition dipole moment of the *n*π_2_^*^ monomer state results in a CD spectrum a negative band at 207 nm, which overlaps with a negative band near 220 nm, which appears as a shoulder. The other modifications lead to computed spectra which are incorrect for an α-helical conformation.

**Table 5:** Inter-amide Interactions (cm^–1^) for Diamide **2l** (−62°, – 41°) α-Helix^a^

method	*n*π_1_^*^-nπ_2_^*^	*n*π_1_^*^-π_nb_π_2_^*^	π_nb_π_1_^*^-*n*π_2_^*^	π_nb_π_1_^*^-π_nb_π_2_^*^
*ab initio*	31	–7	16	–971
diabatic	–359	–660	–131	–363
*n*π_2_^*^	359	–660	131	–363
*n*π_1_^*^	359	660	–131	–363
*n*π_2_^*^ and *n*π_1_^*^	–359	660	131	–363

a The first and second rows are from the DichroCalc *ab initio* and the standard diabatic approaches (as per Rogers et al.^26^), respectively. In the rows that follow, for the state(s) indicated, the monomer electric transition dipole moment has undergone a sign change before diabatization.

The above data suggest that when the angle between the monomer and dimer cross products is greater than 170°, i.e., almost out-of-phase, then our proposed prescription to make the phases consistent, by reversing the sign of the monomer electric transition dipole of the transition on chromophore 2 prior to diabatization, leads to a computed CD spectrum with the correct character. This is most apparent for the diabatic spectrum computed with the modified π_nb_π_2_^*^ monomer state for **2d** (β-strand: −135°, 135°), but it is also evident for the β-strand-like **2f** (data not shown). For the α-helical **2l**, the diabatic spectrum computed with the modified *n*π_2_^*^ monomer state is arguably not an improvement over the standard diabatic spectrum, but is not qualitatively worse.

The closer the diagnostic angle is to 180°, the more likely it is that an inconsistency in the phases has arisen. If the diagnostic angle falls below 170°, this likelihood appears to be much smaller. In our examination of the 13 diamides, each with four couplings, there were five instances where the diagnostic angle was between 160 and 170°: **2a** (π_nb_π_1_^*^-*n*π_2_^*^), **2e** (π_nb_π_1_^*^-*n*π_2_^*^), **2h** (π_nb_π_1_^*^-*n*π_2_^*^), **2j** (π_nb_π_1_^*^-π_nb_π_2_^*^) and **2k** (π_nb_π_1_^*^-π_nb_π_2_^*^). We considered the three most physically relevant of these: **2e** is in the broad β-sheet region of the Ramachandran plot and **2j** and **2k**, which are both α-helical. In each case, application of the modification to the sign of the relevant electric transition dipole moment prior to diabatization led to a computed CD spectrum that was worse than the standard diabatic spectrum. Thus, 170° appears to be the appropriate threshold for the diagnostic angle.

### Example Proteins

We now consider seven example proteins that possess archetypal secondary structure elements and compute their CD using interamide couplings from the diamide diabatization, employing the diabatic couplings with modified monomer π_nb_π_2_^*^ electric transition dipole moment for diamide **2d** (β-strand: −135°, 135°). The seven proteins (and their class) are myoglobin (α helix), jacalin (β strand), concanavalin A (β type I), elastase (β type II), papain (α + β), 3_10_-helix bundle (3_10_-helix) and snow flea antifreeze protein (polyproline helix II (PP II)).

Table 6 lists fractional secondary structure content from DSSP analyses^40^ for entries for exemplar α helix, β strand, β type I, β type II and α + β proteins, whose experimental CD spectra are available in the PCDDB.^41^ In addition, the table shows DSSP analysis for the 3_10_-helix example and the PP II example (from PolyprOnline).^42^

**Table 6:** DSSP Analysis for Exemplar Proteins Myoglobin (α Helix), Jacalin (β Strand), Concanavalin A (β Type I), Elastase (β type II), Papain (α + β) from the PCDDB Entries^a^

protein (PDB entry)	α helix	3_10_ helix	π helix	β strand	β bridge	bonded turn	bend	loop or irregular	PP II
myoglobin (1ymb)	0.739	0.000	0.000	0.000	0.000	0.131	0.020	0.111	n/a
jacalin (1ku8)	0.000	0.000	0.000	0.626	0.007	0.056	0.088	0.224	n/a
concanavalin A (1nls)	0.0	0.038	0.0	0.460	0.008	0.118	0.135	0.241	n/a
elastase (3est)	0.054	0.042	0.0	0.304	0.017	0.138	0.054	0.392	n/a
papain (1ppn)	0.231	0.028	0.000	0.179	0.042	0.132	0.104	0.283	n/a
3_10_-helix bundle (7qdi)	0.021	0.891	0.0	0.0	0.0	0.004	0.0	0.080	n/a
snow flea antifreeze protein (2pne)	0.0	0.0	0.0	0.0	0.049	0.049	0.123	0.062	0.716

a 3_10_-helix bundle (3_10_-helix) from analysis using DSSP. Snow flea anti-freeze protein (PP II) DSSP-PPII analysis from the PolyprOnline database.

Table 7 lists the number of instances where a diamide (ϕ, ψ) angle pair is used for each of the seven proteins to compute the CD. For the β-rich proteins, the PP II diamide geometry **2m** (−75°, 145°) is frequently used.

**Table 7:** Number of Chains, Residues (Dihedral Pairs) and Instances Where a Diamide (ϕ, ψ) Pair is Used for Each of the Seven Proteins to Compute the CD^a^

	myoglobin (1ymb)	jacalin (1ku8)	concanavalin A (1nls)	elastase (3est)	papain (1ppn)	3_10_-helix bundle (inverted 7qdi)	snow flea antifreeze protein (2pne)
chains	1	8	1	1	1	8	1
residues (dihedral pairs)	153 (151)	596 (587)	237 (235)	240 (238)	212 (210)	238 (229)	81 (79)
**2a** (180, 180)	0	14	0	2	5	7	0
**2b** (−120, 180)	0	41	16	16	10	0	1
**2c** (−60, 180)	1	2	6	5	1	0	4
**2d** (−135, 135)	0	147	82	28	28	0	3
**2e** (−120, 120)	4	108	27	28	11	0	2
**2f** (−120, 60)	4	8	5	8	8	1	2
**2g** (−120, 0)	3	18	9	12	16	3	1
**2h** (−60, 0)	4	12	12	11	10	12	4
**2i** (−74, −4)	17	25	19	21	13	81	0
**2j** (−48, −57)	20	47	5	10	14	1	2
**2k** (−60, −60)	18	29	3	8	12	0	0
**2l** (−62, −41)	72	24	12	30	50	123	0
**2m** (−75, 145)	8	112	39	59	32	1	60

a PDB entry in parentheses. ϕ and ψ angles in degrees.

### Computed CD Spectra

**Figure 4:**
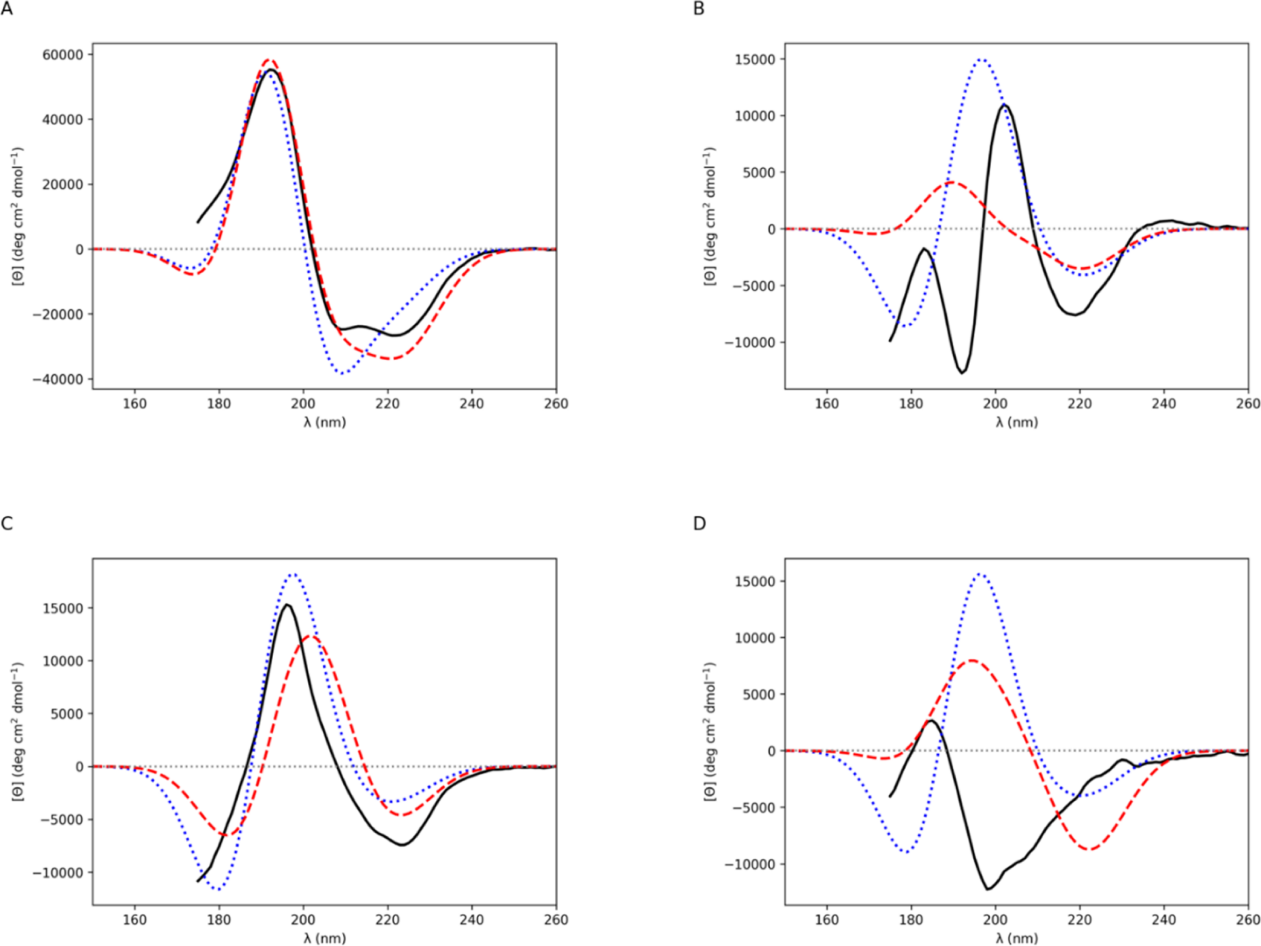
Computed CD spectra for (A) myoglobin (α helix), (B) jacalin (β strand), (C) concanavalin A (β type I) and (D) elastase (β type II). Gaussians of fwhm of 12.5 nm were fitted to the rotational strength line spectra to obtain the CD spectra. Experimental (solid black line), *ab initio* NMA4FIT2 (dotted blue line), and diabatic with modified **2d** π_nb_π_2_^*^ electric transition dipole moment (dashed red line).

**Figure 5:**
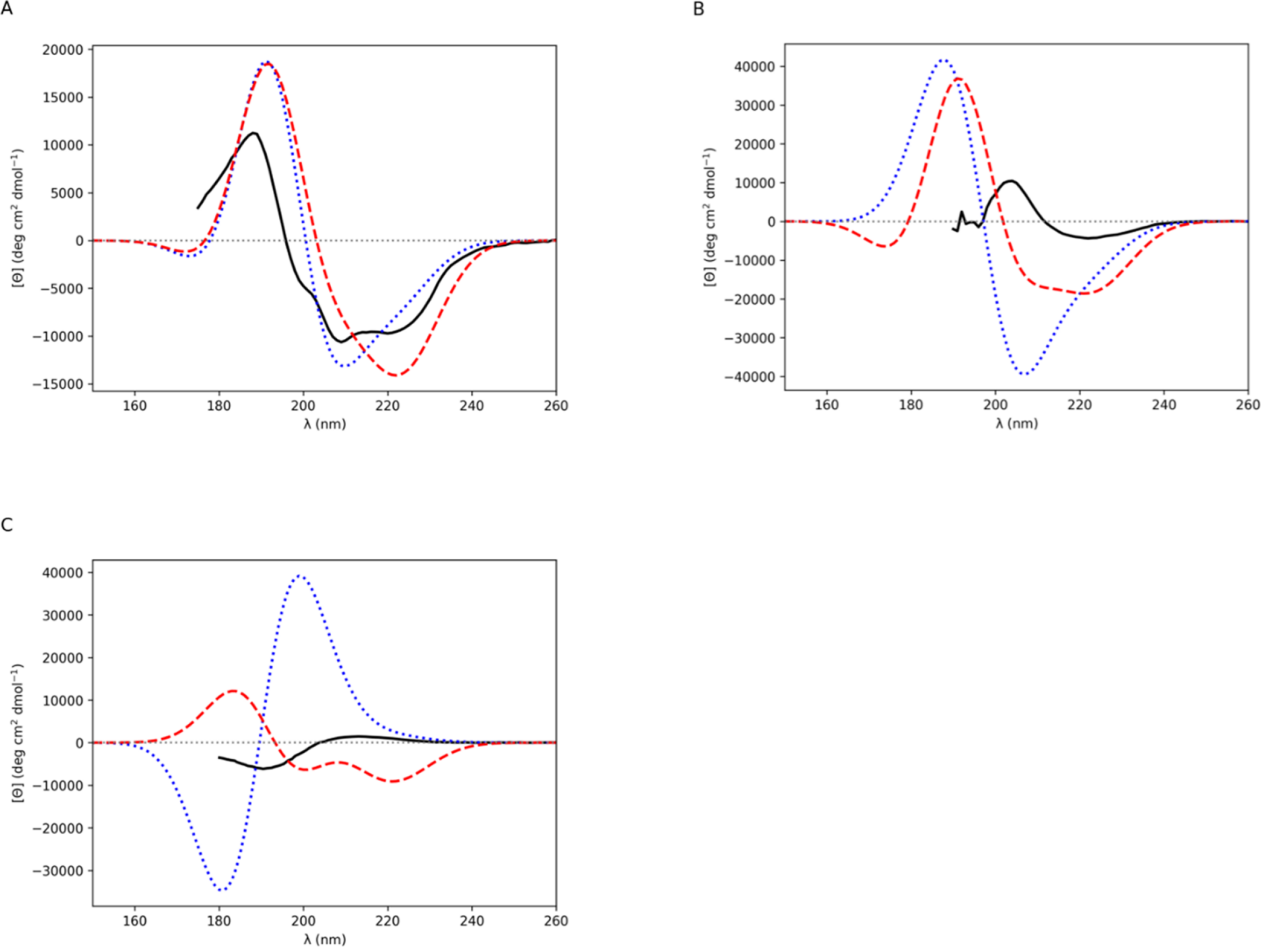
Computed CD spectra for (A) papain (α + β), (B) 3_10_-helix bundle (3_10_-helix) and (C) snow flea antifreeze protein (PP II). Gaussians of fwhm of 12.5 nm were fitted to the rotational strength line spectra to obtain the CD spectra. Experimental (solid black line), *ab initio* NMA4FIT2 (dotted blue line), and diabatic with modified **2d** π_nb_π_2_^*^ electric transition dipole moment (dashed red line).

Figures 4 and 5 display experimental CD spectra and CD spectra computed for each of the example proteins.

### α Helix

Myoglobin is a soluble globular α–helical protein. PDB entry 1ymb(43) has a DSSP value for α helix = 0.739. The experimental (from the PCDDB^41^) and computed CD spectra are shown in Figure 4A. Application of the diabatic couplings gave no ϕ and ψ angle pairs closest to diamide **2d** (−135°, 135°) (Table 7); therefore, the standard diabatic spectrum is identical to the modified diabatic spectrum for this case. The experimental spectrum for an α helical protein, such as myoglobin, features an intense positive band peak at 190 nm and a double minimum band with the negative band peaks at 208 and 220 nm. For myoglobin, these peaks are observed experimentally at 192, 209, and 221 nm. The *ab initio* and the diabatic computed spectra are both in line with experiment in predicting the intense positive band peak at 191 and 192 nm, respectively (Figure 4A). The *ab initio* spectrum has a single negative band minimum at 209 nm whereas the diabatic spectrum has a broad negative band with a minimum at 221 nm and an intense band shoulder at around 210 nm.

### β Strand

Based on their CD spectra, proteins rich in β-strands can be separated into two distinct classes: β type I and β type II.^44,45^ β-I proteins, e.g., concanavalin A, contain regular β strands and yield CD spectra with a positive band at 195 nm and a negative band at 215–220 nm. β-II proteins, e.g., elastase, give rise to CD spectra reminiscent of unordered polypeptides with a negative band at around 198 nm.^46^ β-II proteins may adopt a more polyproline II like conformation,^47^ in addition to the possibility that they are more conformationally labile. Three β-rich proteins are considered herein.

Jacalin (agglutanin) is a soluble globular β protein. PDB entry 1ku8(48) has a DSSP value for β strand = 0.626. The experimental (from the PCDDB^41^) and computed CD spectra are shown in Figure 4B. Application of the diabatic couplings gave the largest absolute number of ϕ and ψ angle pairs (147) closest to diamide **2d** (−135°, 135°) (Table 7). The *ab initio* and the diabatic computed spectra are both in line with experiment in predicting the negative band peak observed at 219 nm, albeit red-shifted; the *ab initio* is at 221 nm and the diabatic is at 220 nm (Figure 4B). At shorter wavelengths, the agreement with experiment differs, where the experimental spectrum has a positive band peak at 202 nm and a negative band peak at 192 nm. The *ab initio* spectrum has a positive band peak at 197 nm and a negative band peak at 179 nm. The diabatic spectrum features a less intense band maximum at 190 nm and a very shallow band minimum at 171 nm.

Concanavalin A is a β type I protein. PDB entry 1nls(49) has a DSSP value for β strand = 0.46. The experimental (from the PCDDB^41^) and computed CD spectra are shown in Figure 4C. Application of the diabatic couplings gave 82 ϕ and ψ angle pairs closest to diamide **2d** (−135°, 135°), corresponding to approximately one-third of the residues (Table 7). The *ab initio* and the diabatic computed spectra broadly concur with experiment. They both predict the negative band peak observed at 223 nm, where the *ab initio* computed spectral peak is shifted slightly to the blue at 221 nm (Figure 4C), in addition to the positive band peak observed at 196 nm and the negative band observed at around 175 nm. However, both computed spectra are red-shifted with peaks at 197 and 180 nm (*ab initio*) and at 202 and 182 nm (diabatic).

Elastase is a β type II protein. PDB entry 3est(50) has a DSSP value for β strand = 0.304. The experimental (from the PCDDB^41^) and computed CD spectra are shown in Figure 4D. Application of the diabatic couplings gave 28 ϕ and ψ angle pairs closest to diamide **2d** (−135°, 135°) (Table 7). The experimental spectrum possesses a weak positive peak at 185 nm and a negative band peak at 198 nm with a broad negative band shoulder that becomes less intense as wavelength increases (Figure 4D). The *ab initio* spectrum features a negative band peak at 179 nm, a positive band peak at 196 nm and a negative band minimum at 220 nm. The diabatic spectrum has a positive band peak at 194 nm and a negative band peak at 222 nm (resembling a couplet centered at 209 nm) and a very shallow negative band with a peak at 173 nm.

### α + β

Papain is a soluble globular α + β protein. PDB entry 1ppn(51) has a DSSP value for α helix = 0.231 and β strand = 0.179. The experimental (from the PCDDB^41^) and computed CD spectra are shown in Figure 5A. Application of the diabatic couplings gave 28 ϕ and ψ angle pairs closest to diamide **2d** (−135°, 135°) (Table 7). The experimental spectrum of this α + β protein (Figure 5A) combines characteristics of the spectrum for the α helix protein, myoglobin (Figure 4A), and the spectrum for the β type I protein, elastase (Figure 4D). The observed spectrum for papain (Figure 5A) possesses a positive peak at 188 nm and a negative band peak at 209 nm with a broad negative band shoulder at 220 nm. The *ab initio* spectrum features a shallow negative band peak at 173 nm, a positive band peak at 191 nm and a negative band minimum at 210 nm with a broad negative band shoulder that becomes less intense as wavelength increases. The diabatic spectrum has a shallow negative band peak at 172 nm, a positive band peak at 192 nm and a negative band minimum at 222 nm with a narrow negative band shoulder at around 209 nm.

### 3_10_-Helix

The octameric left-handed 3_10_-helix bundle is a 3_10_-helical protein. PDB entry 7qdi(52) has a DSSP value for 3_10_ helix = 0.891. The experimental CD spectrum is from Kumar et al.^52^ The PDB coordinates were inverted, for the CD computations, so that the protein is the right-handed form for natural l-amino acids (the left-handed form comprises d-amino acids). The experimental CD was obtained at 100 μM concentration, and its sign inverted for comparison with the computed CD. The experimental and computed CD spectra are shown in Figure 5B. There are no ϕ and ψ angle pairs closest to diamide **2d** (−135°, 135°) (Table 7); therefore, the standard diabatic spectrum is identical to the modified diabatic spectrum for this case. The experimental spectrum (Figure 5B) possesses a positive band peak at 204 nm and a broad and shallow negative band with a minimum at 222 nm. The *ab initio* spectrum resembles a couplet centered at 198 nm with a positive band peak at 188 nm and a negative band peak at 207 nm, with a negative band shoulder at around 220 nm. The diabatic spectrum resembles that predicted for an α-helical protein, with a positive band peak at 191 nm and a broad negative band with a minimum at 221 nm and an intense band shoulder at around 210 nm.

### PP II

The snow flea antifreeze protein is a PP II rich protein. PDB entry 2pne(53) has a DSSP-PPII value for PP II = 0.716. The experimental CD spectrum is Figure S1 from the study by Graham and Davies.^54^ The experimental and computed CD spectra are shown in Figure 5C. Application of the diabatic couplings gave three ϕ and ψ angle pairs closest to diamide **2d** (−135°, 135°) (Table 7). The experimental spectrum has a negative band with a minimum at 190 nm and a weak and broad positive band with a maximum at 212 nm (Figure 5C). The *ab initio* spectrum resembles a couplet centered at 190 nm with a negative band peak at 181 nm and a positive band peak at 199 nm, with a weak and positive band shoulder at around 220 nm. The diabatic spectrum, in contrast, features a positive band peak at 183 nm and a double minimum with the two negative peaks at 200 nm and at 221 nm (as predicted for an α-helical protein).

To quantify the difference in accuracy between the *ab initio* and the diabatic-computed spectra, the mean absolute error (MAE) with experimental spectra were evaluated over the wavelength range 190 to 240 nm and the span of the range of the experimental spectra. Table S4 displays the MAE of the spectra computed for each of the seven example proteins. Diabatic spectra for five of the seven proteins have a smaller MAE compared to the *ab initio* spectra over the 190 to 240 nm range; two proteins (concanavalin A and papain) have *ab initio* spectra with smaller MAE compared to the diabatic spectra. Over the span of the range of the experimental spectra, the MAE for the *ab initio* computed spectrum for jacalin is slightly smaller than for the diabatic spectrum (Table S4).

## Discussion

The diabatic computed CD spectrum for the α–helical protein myoglobin is in very good agreement with experiment (Figure 4A). It possesses a broad negative band with a minimum at 221 nm and an intense band shoulder at around 210 nm, which is characteristic of an α–helical protein. The density of (ϕ, ψ) angles for this protein is clustered (Figure S1A) in the 3_10_-helical and α-helical region of the Ramachandran plot, i.e., diamide geometries **2i** (−74°, −4°), and **2j** (−48°, −57°), **2k** (−60°, −60°) and **2l** (−62°, −41°).

For the β-rich proteins, jacalin, concanavalin A, elastase and papain, the PP II diamide geometry **2m** (−75°, 145°) is frequently used to compute the diabatic spectra (Table 7). The Ramachandran plots are shown in Figure S1(B–E) where it is clear that each of these four proteins have (ϕ, ψ) angles clustered around the diamide geometry **2m** (−75°, 145°). Moreover, all four of these protein structures have relatively large proportions of loop or irregular structures from the DSSP analyses (Table 6) with elastase having the largest proportion (0.392). For elastase, 25% of the residues are assigned the **2m** (−75, 145°) PP II diamide geometry (Table 7). This may account for why the band features of the experimental spectrum are not predicted by either the *ab initio* spectrum nor the diabatic spectrum (Figure 4D). This suggests that the interamide couplings underlying the CD for a protein with secondary structure in the region of the Ramachandran plot around the **2m** (−75°, 145°) PP II diamide geometry warrant further investigation. As discussed above, β type II proteins, such as elastase, give rise to CD spectra reminiscent of unordered polypeptides with a negative band at around 198 nm,^46^ and may adopt a more PP II like conformation,^47^ in addition to the possibility that they are more conformationally labile.

An analysis of molecular dynamics (MD) simulations on concanavalin A (β-I type) and elastase (β-II type) shows that, during the course of the 20 ns simulations, elastase has more PP II secondary structure than concanavalin A and that concanavalin A has more β-sheet secondary structure than elastase. Figure S2 shows a Ramachandran plot of the difference between ϕ and ψ dihedral angles for elastase with concanavalin A using histograms of the dihedral angles obtained from snapshots taken every 50 ps from the MD simulations. Along with the analysis of the crystal structures (Tables 6 and 7), the MD analysis helps verify the contention that β II proteins adopt more PP II conformation and goes some way to confirming the speculation that the distinction between the two classes of β rich proteins can be made based on their relative contents of PP II and β sheet structure. Hirst et al.^46^ computed the CD spectra for concanavalin A and elastase using structural snapshots from generalized Born MD simulations (sampled every 0.5 ps from 400 ps production dynamics at 298 K). The mean CD spectrum computed for the ensemble of concanavalin A remained close to that computed for the X-ray crystal structure used in the study, whereas the ensemble of elastase gave a mean computed CD spectrum that was significantly different from that of the X-ray crystal structure. The mean CD spectrum for elastase was an improvement despite the sign of the peak being wrong at 195 nm. A relaxation of the elastase structure in solution may account for some of the previously unexplained differences between β-I and β-II proteins. From an analysis of the backbone dihedral angles from the MD trajectories for concanavalin A and elastase, it was observed that a shift in the dihedral angle population from (−85°, 95°) to (−70°, 140°), i.e., to more PP II-like, significantly influenced the computed CD, bringing the calculated CD spectrum for elastase closer to the experimentally observed spectrum.

**Figure 6:**
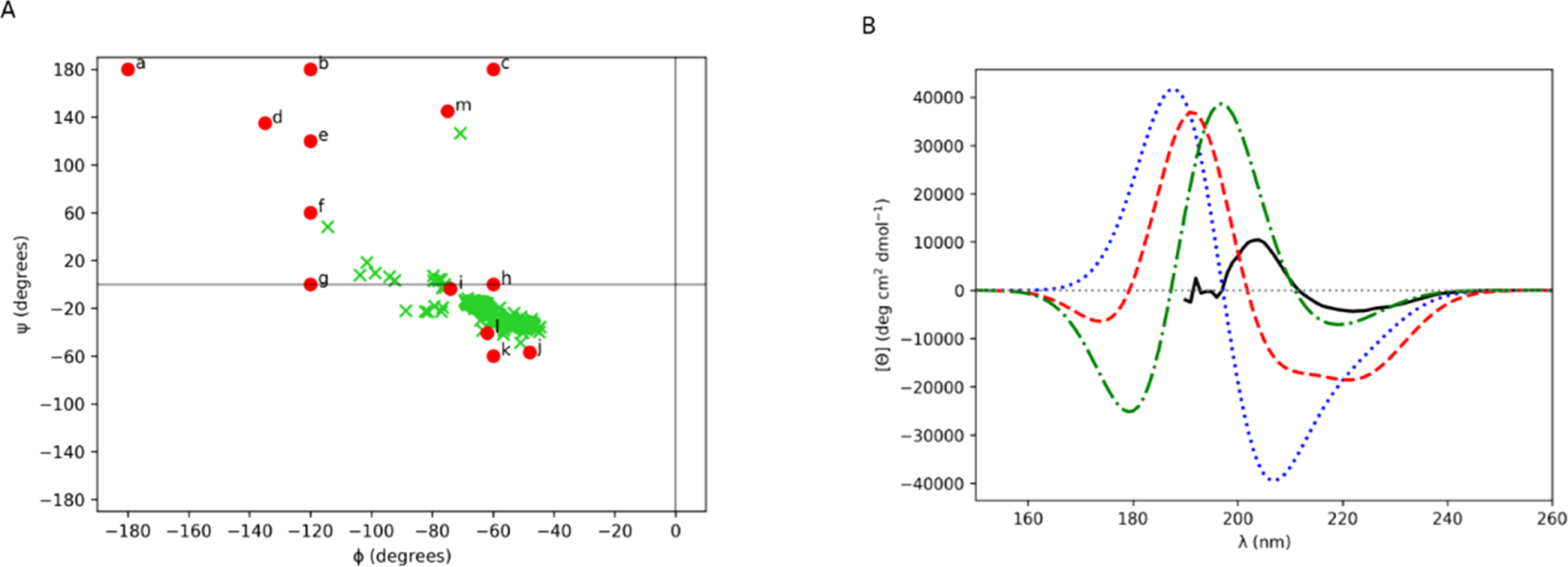
(A) Ramachandran plot for 3_10_-helix bundle (lime green crosses) on which the 13 diamide (ϕ, ψ) angles are superimposed (red dots and diamide labeled by its letter). (B) Computed CD spectra for 3_10_-helix bundle (3_10_-helix). Gaussians of fwhm of 12.5 nm were fitted to the rotational strength line spectra to obtain the CD spectra. Experimental (solid black line), *ab initio* NMA4FIT2 (dotted blue line), diabatic with modified **2d** π_nb_π_2_^*^ electric transition dipole moment (dashed red line), and all **2l** (−62°, −41°) interamide couplings substituted for **2i** (−74°, −4°) interamide couplings in the Hamiltonian (dash-dotted green line).

For the 3_10_ helix bundle the α-helical diamide geometry **2l** (−62°, −41°) is used more often than the 3_10_-helical diamide geometry **2i** (−74°, −4°) (Table 7), and this may give rise to the computed diabatic spectrum that resembles the spectrum for an α-helical protein (Figure 5B). The Ramachandran plot for the inverted 3_10_ helix bundle is displayed in Figure 6A on which the 13 diamide (ϕ, ψ) angles are superimposed. The plot shows that a significant proportion of the biomolecule’s (ϕ, ψ) angles lie between the 3_10_ helix diamide geometry **2i** (−74°, −4°) and the three α-helical diamide geometries **2j** (−48°, −57°), **2k** (−60°, −60°) and **2l** (−62°, −41°). Substituting all the 123 **2l** (−62°, −41°) interamide couplings for the **2i** (−74°, −4°) interamide couplings in the Hamiltonian, yields a diabatic spectrum that possesses a relatively weak negative band peak at 219 nm, a positive band peak at 197 nm and a negative band peak at 179 nm (Figure 6B). This spectrum is in much better agreement with experiment, in particular the negative band at around 220 nm. The MAE for the *ab initio*, the diabatic and the **2l** to **2i** substituted diabatic spectra over the wavelength range 190 to 260 nm are, respectively, 14240.5, 11184.6, and 6598.7 deg cm^2^ dmol^–1^ (Table S4). The interamide couplings *n*π_1_^*^-*n*π_2_^*^, *n*π_1_^*^-π_nb_π_2_^*^, π_nb_π_1_^*^-*n*π_2_^*^ and π_nb_π_1_^*^-π_nb_π_2_^*^ for diamide **2i** (−74°, −4°) are 289, −148, 230, and 284 cm^–1^; and, for diamide **2l** (−62°, −41°) are −359, −660, −131, and −363 cm^–1^.^26^ The *n*π_1_^*^-π_nb_π_2_^*^ interamide coupling reduces in magnitude for diamide **2l** to diamide **2i** with the remaining three couplings of similar magnitude with a change in sign for diamide **2l** to diamide **2i**. The *n*π_1_^*^-π_nb_π_2_^*^ interamide coupling for a diamide is described by the **μ-*****m*** mechanism (dipole–quadrupole coupling),^55^ that has a relatively large magnitude from the diabatization procedure for the three α-helical diamide geometries **2j** (−48°, −57°), **2k** (−60°, −60°) and **2l** (−62°, −41°).^26^

The *ab initio* and the diabatic computed CD spectra for the PP II example, snow flea antifreeze protein, are both in poor agreement with experiment (Figure 5C). The Ramachandran plot (Figure S1G) shows the biomolecule’s (ϕ, ψ) dihedral angles clustered around PP II diamide geometry **2m** (−75°, 145°). This suggests that further investigation of the interamide couplings underlying the spectrum of a PP II protein, around diamide geometry **2m** (−75°, 145°), may help improve the diabatization procedure for polypeptides that adopt this secondary structure arrangement (as discussed above for the β type II protein elastase).

Woody computed the CD spectra for Ala_20_ polypeptides in two PP II secondary structural arrangements and Pro-containing peptides in the PP II conformation using a methodology based on the polarizability of groups of atoms to include deep-UV electronic transitions. These higher-energy transitions were mixed with the *n*π* (at 220 nm), NV_1_ (π_nb_π* at 190 nm) and NV_2_ (π_b_π* at 140 nm) transitions of an amide group in exciton theory calculations.^56^ The CD spectrum computed for Ala_20_ (−60°, 160°), incorporating the polarizability terms, features a weak positive band near 220 nm and a strong negative band near 200 nm that are in excellent agreement with the experimental spectrum of poly(Glu) in water (considered as a model for a PP II helix of poly(Ala)). Thus, it is important to consider the mixing of higher energy transitions with the *n*π* and π_nb_π* transitions of the amide group when computing the CD of a PP II structure. Woody’s methodology was applied to compute the CD and help characterize the intermolecular structures of β_2_-microglobulin (β_2_m) core fragments in amyloid fibrils in which backbone conformations and aromatic side chain conformations affected the CD.^57^

## Conclusions

The CD spectra computed using the interamide couplings from the diabatization procedure have been shown to yield improved spectra, with the exception of β-rich concanavalin A and papain, over that of the CD spectra computed using the *ab initio* parameters. The comparison of CD spectra and of secondary structural assignments have shown that additional diamide geometries to span the Ramachandran plot around and between, e.g., diamides **2d** (−135°, 135°) and **2m** (−75°, 145°), and diamides **2i** (−74°, −4°) and **2l** (−62°, −41°), may enhance the computed CD spectra obtained from the diabatization methodology outlined in this study.

## Supporting Information Available

A segment from an example DichroCalc Hamiltonian; Cross products used to calculate the optical factors; locations of the reference oxygen atoms used to compute the vectors between neighboring amide chromophores; Mean absolute errors; Ramachandran plots for the seven example proteins on which the 13 diamide (ϕ, ψ) angles are superimposed. A Ramachandran plot of the difference between f and ψ dihedral angles for elastase with concanavalin A from analysis of MD simulations. The DichroCalc code is available at https://github.com/dmrogers75/DichroCalc. Data, example files and other code used in this study are available in a folder named “diabatisation” (PDF)

## Abbrreviations

CASSCF: complete active space self-consistent field
CD: electronic circular dichroism
NAGMA: *N*-acetylglycine *N*′-methylamide
PDB: Protein Data Bank
UV: ultraviolet
PP II: polyproline helix II
MAE: mean absolute error
TDDFT: time-dependent density functional theory
MD: molecular dynamics
