# Supplementary material for: An Improved Diabatization Scheme for Computing the Electronic Circular Dichroism of Proteins
Source: J Phys Chem B. 2024 Jul 22;128(30):7350–61. doi: 10.1021/acs.jpcb.4c02582 (PMC11301672; doi:10.1021/acs.jpcb.4c02582)
Supplement: Supplementary file 1 — jp4c02582_si_001.pdf [file jp4c02582_si_001.pdf]

An Improved Diabatization Scheme for Computing the Electronic Circular Dichroism of Proteins

David M. Rogers[a], Hainam Do[b] and Jonathan D. Hirst[a]*

[a]School of Chemistry, University of Nottingham, University Park, Nottingham, NG7 2RD United Kingdom

[b]Department of Chemical and Environmental Engineering and Key Laboratory of Carbonaceous Waste Processing and Process Intensification Research of Zhejiang Province, University of Nottingham Ningbo China, Ningbo 315100, China; New Materials Institute, University of Nottingham Ningbo China, Ningbo 315042, China

[*]jonathan.hirst@nottingham.ac.uk

SUPPLEMENTARY INFORMATION

Table S1 displays a segment from the DichroCalc Hamiltonian matrix for myoglobin before and after modification. The cross products used to calculate the optical factors are in Table S2 along with the locations of the reference oxygen atoms, in Table S3, used to compute the vectors between neighboring amide chromophores. Table S4 displays mean absolute error (MAE) of computed spectra with experimental spectra for the seven example proteins. Ramachandran plots for the seven example proteins on which the 13 diamide (ϕ, ψ) angles are superimposed are in Figure S1. Figure S2 shows a Ramachandran plot of the difference

between ϕ and ψ dihedral angles for elastase with concanavalin A using histograms of the dihedral angles obtained from snapshots taken every 50 ps from the molecular dynamics (MD) simulations. The MD simulations on concanavalin A and elastase were performed at equilibrium conditions at 298 K using the CHARMM36 force field with TIP3P to describe water. Production dynamics was carried out for 20 ns and structural snapshots were extracted from the trajectories every 50 ps.

45455.000000	82.376413	31.541026	-24.151603	0.412920	-2.743926
	51813.000000	82.276016	-927.777039	27.029457	-145.993713
		45455.000000	147.114592	14.760797	-31.750347
			51813.000000	-39.550625	-908.573605
				45455.000000	33.281737
					51813.000000
45455.000000	82.376413	80.898600	356.368800	0.412920	-2.743926
	51813.000000	145.090000	29.222600	27.029457	-145.993713
		45455.000000	147.114592	80.898600	356.368800
			51813.000000	145.090000	29.222600
				45455.000000	33.281737
					51813.000000

Table S1. Standard DichroCalc Hamiltonian matrix (upper) and modified DichroCalc Hamiltonian (lower) for the first three peptide bonds in myoglobin (PDB entry 1ymb). The matrix elements have units of cm^{-1}. The inter-amide couplings were replaced by diabatic couplings from diamide **2m** (-75°, 145°) and are shown in red text in the modified Hamiltonian.

Diamide	μ_i - μ_j	Monomer / dimer cross product components (au)
**2a**	1-3	-4.18533125e-05 -1.08196447e-05 7.25599098e-07<br>1.22683961e-04 -2.82171717e-04 2.87784072e-05
	1-4	-0.07467305 -0.06903676 0.00436714<br>0.08999541 0.02909727 -0.00570739
	2-3	0.05576023 -0.08496872 0.00570168<br>-0.03874081 0.09479203 -0.00956834
	2-4	-0.00169699 0.08496085 1.28951289<br>-0.00408667 0.15065169 2.27604197
**2b**	1-3	-0.00381651 0.00470141 -0.00056675<br>-0.00110617 0.00370057 -0.00053508
	1-4	-0.05660111 -0.06482121 -0.05311526<br>-0.04889589 -0.01055435 -0.10983567
	2-3	0.03338267 -0.05557902 0.00892541<br>0.02541554 -0.07994812 0.01208777
	2-4	-0.51366275 -0.47942685 1.0788413<br>-0.93760657 -0.72033681 1.7339161
**2c**	1-3	0.00590166 0.0041791 -0.00274886<br>-0.00107613 -0.00638322 0.0011084
	1-4	0.00699617 0.05317995 0.08426049<br>-0.03977326 0.08311854 0.04143086
	2-3	0.03449576 -0.01268639 -0.00141127<br>-0.02680906 0.05376414 -0.02925038
	2-4	0.62006279 -0.92072504 0.60860801<br>1.12232913 -1.33718914 0.69561444
**2d**	1-3	0.00089999 0.00603109 -0.00011984<br>0.00126758 0.00367665 -0.00102234
	1-4	-0.07229225 -0.03495884 -0.03139863<br>0.05257908 0.06069488 -0.00438034
	2-3	0.0611685 -0.05384251 -0.03038675<br>0.08933123 -0.04422156 -0.01444271
	2-4	-0.06850373 -0.44058191 1.19147384<br>0.09241983 0.47375492 -1.64104543
**2e**	1-3	0.00073837 0.00670444 -0.0017708<br>-0.00138848 0.0068046 -0.00425507
	1-4	-0.07501013 -0.0293059 -0.03711468<br>0.03081538 0.05380097 -0.00545668
	2-3	0.06526051 -0.04596078 -0.03067021<br>-0.13215728 0.04010281 0.06550332
	2-4	-0.08838841 -0.10309095 1.13161174<br>-0.08612881 0.03332493 1.64181203
**2f**	1-3	-0.00610654 -0.00396622 -0.00233929<br>-0.00143129 -0.00145609 -0.00390139

	1-4	0.04367524 0.00237822 -0.05512045<br>-0.023151 0.00093478 0.0319315
	2-3	-0.0571981 0.07756828 -0.00911831<br>-0.075888 0.09036748 -0.02563899
	2-4	-0.63580387 -0.01914832 0.70298571<br>1.1359466 -0.14241738 -0.66011353
**2g**	1-3	0.00610034 -0.00386658 -0.0006167<br>0.00076274 0.00179659 0.00125935
	1-4	0.04935934 0.01025354 0.04966298<br>-0.11650409 -0.01866497 -0.03152387
	2-3	-0.00887531 0.05573351 -0.00445702<br>0.06127032 -0.0414444 -0.04837788
	2-4	0.94573277 0.0358107 -0.90626828<br>-1.63858666 -0.38985654 1.52167066
**2h**	1-3	-0.00615081 -0.00315585 0.00140012<br>-0.0038191 -0.00031425 0.00193928
	1-4	0.03598201 -0.03066812 -0.06643105<br>0.08107096 -0.02690378 -0.05683482
	2-3	-0.05690696 0.01872431 0.00198826<br>0.07227059 0.00048352 0.00803359
	2-4	-0.99765429 0.35697956 0.00239224<br>-1.49960611 0.70981046 -0.24389233
**2i**	1-3	-0.00657877 -0.0029841 0.00248964<br>-0.00349701 -0.00062414 0.0034163
	1-4	0.00829795 -0.02720017 -0.06793186<br>0.00468713 -0.03369844 -0.05669257
	2-3	-0.03328783 0.01255671 0.00470307<br>-0.0588759 0.00487177 0.02109771
	2-4	7.16438595 2.7879867 1.79866202<br>1.70413125 -0.8325204 -0.07642202
**2j**	1-3	-0.00576552 -0.0002717 0.00539467<br>0.00095591 -0.00012353 0.00011881
	1-4	-0.02004528 -0.026988 -0.02764066<br>-0.09275878 0.01124965 -0.00220946
	2-3	0.0164087 -0.03542428 0.02059528<br>-0.02675736 -0.00018668 0.01206859
	2-4	-1.27808435 0.05080355 0.20115334<br>1.78044729 0.00489123 0.06625706
**2k**	1-3	0.00497688 -0.00244777 -0.0054704<br>-0.000566358682 -0.0000286777996 -0.000191239504
	1-4	0.04026439 -0.02262578 0.02532069<br>0.05942664 -0.0036452 0.00197494
	2-3	-0.0361258 -0.03240907 0.00363621<br>-0.11643769 0.00812357 -0.00994725

	2-4	1.17953175 -0.06164057 -0.32284028<br>-1.37573062 0.12924579 -0.11670288
**2l**	1-3	-0.0059695 -0.00143828 0.00496088<br>1.63330656e-03 9.26084902e-05 -1.52671207e-03
	1-4	-0.02261393 -0.02895643 -0.04293952<br>0.06319306 0.00306987 0.00451803
	2-3	0.00879538 -0.02610088 0.01144515<br>-0.11818146 0.02648671 0.00289068
	2-4	-1.26370514 0.23326444 0.20791987<br>-1.60788544 0.19679628 -0.18973654
**2m**	1-3	0.00499655 -0.00497532 -0.00356633<br>0.00329044 -0.00165259 -0.0086432
	1-4	0.05033729 0.07902501 -0.03977498<br>-0.08034015 0.05089746 -0.15122326
	2-3	-0.02292799 0.0002196 -0.02584597<br>0.00135002 0.02238281 0.06780755
	2-4	0.74316337 0.02619018 0.98393251<br>1.30159762 -0.04091806 1.26888467

Table S2. Cross product ($\boldsymbol{\mu}_j \times \boldsymbol{\mu}_i$) Cartesian components (atomic units) for the inter-amide couplings for each diamide for the monomer and dimer transition dipole moments. The 1-3, 1-4, 2-3 and 2-4 notation describe, respectively, the $n\pi_1^* - n\pi_2^*$, $n\pi_1^* - \pi_{nb}\pi_2^*$, $\pi_{nb}\pi_1^* - n\pi_2^*$ and $\pi_{nb}\pi_1^* - \pi_{nb}\pi_2^*$ couplings.

**Diamide**	**Reference oxygen atom position (angstroms) for amide 1 / amide 2**
**2a**	2.611657 1.360162 -0.082226<br>-1.364712 -1.356210 0.092209
**2b**	2.333689 1.351044 0.489743<br>-1.156560 -1.324595 0.131890
**2c**	-1.511516 -0.204171 -1.433906<br>0.623022 -0.888790 0.712523
**2d**	2.355410 1.425501 0.071999<br>-1.496493 -1.440329 -0.318648
**2e**	2.116466 1.414748 -0.337838<br>-1.618576 -1.527482 0.084758
**2f**	-2.004731 -0.563667 -1.236570<br>2.371757 1.315144 0.312169
**2g**	2.132148 0.680754 1.151056<br>-2.398275 1.336162 -0.123800
**2h**	-1.309051 -0.583084 -1.216912<br>2.433967 1.128048 -0.200235
**2i**	-1.406260 -0.376723 -1.336337<br>2.384962 1.098613 -0.384528
**2j**	-1.044455 -0.419284 -1.208108<br>1.834160 1.031165 -0.989208
**2k**	1.231938 0.063284 1.300863<br>-1.748517 1.245609 0.778501
**2l**	-1.198294 -0.249723 -1.325119<br>2.011239 1.047258 -0.836990
**2m**	-1.451674 -1.452917 -0.332212<br>1.054693 1.068483 0.991825

Table S3. Reference oxygen atom Cartesian coordinates (angstroms) for monomer and dimer transition dipole moments. Reference atom for amide 1 and for amide 2. $\boldsymbol{r}_{ji} = \boldsymbol{r}_j - \boldsymbol{r}_i$.

**Protein (PDB entry)**	**Wavelength range (nm)**	***Ab initio***	**Diabatic**
Myoglobin (1ymb)	190 – 240<br>175 – 279	7743.4<br>4917.0	4600.4<br>3685.0
Jacalin (1ku8)	190 – 240<br>175 – 279	5195.2<br>3219.5	4819.9<br>3480.2
Concanavalin A (1nls)	190 – 240<br>175 – 279	3335.4<br>2074.0	4064.9<br>2529.2
Elastase (3est)	190 – 240<br>175 – 277	8572.7<br>5215.8	7782.5<br>4224.8
Papain (1ppn)	190 – 240<br>175 – 279	3628.9<br>2359.0	5497.5<br>3175.2
3_{10}-helix bundle (inverted 7qdi)	190 – 240<br>190 – 260	19784.1<br>14240.5	15409.1 (9169.2)<br>11184.6 (6598.7)
Snow flea antifreeze protein (2pne)	190 – 240<br>180 – 260	16021.5<br>13161.1	5566.5<br>5912.0

Table S4. Mean absolute error (deg cm^2 dmol^{-1}) of computed spectra with experimental spectra for the seven example proteins. Wavelength range of 190 nm to 240 nm and the span of the range of the experimental spectra. For the 3_{10}-helix bundle, the MAE for substituting all the 123 **2l** (-62°, -41°) inter-amide couplings for the **2i** (-74°, -4°) inter-amide couplings are in parentheses.

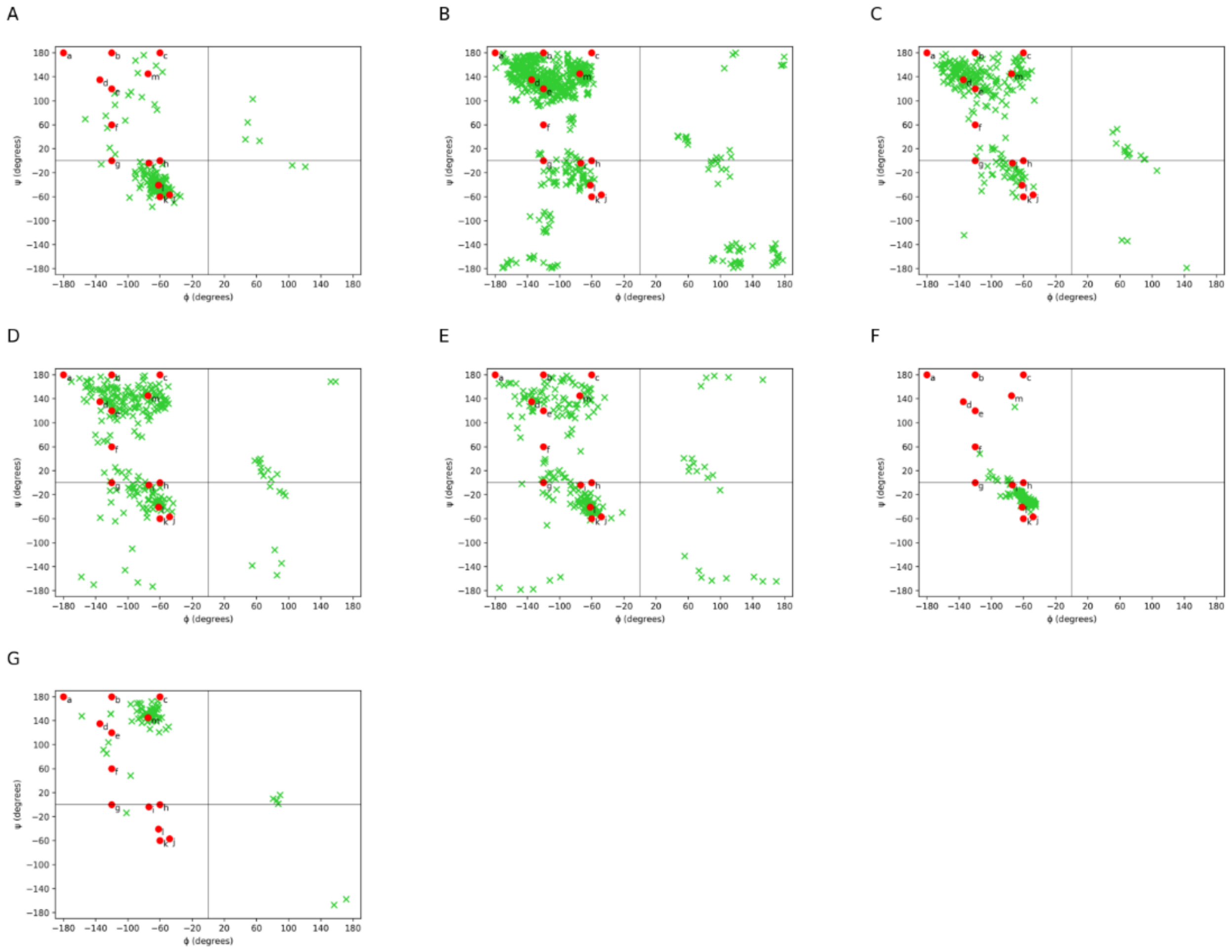

Figure S1. Ramachandran plots for the seven example proteins (lime green crosses) on which the 13 diamide (ϕ, ψ) angles are superimposed (red dots and diamide labelled by its letter). (A) myoglobin (α helix, 1ymb), (B) jacalin (β strand, 1ku8), (C) concanavalin A (β type I, 1nls), (D) elastase (β type II, 3est), (E) papain (α+β, 1ppn), (F) 3_{10}-helix bundle (3_{10}-helix, inverted 7qdi) and (G) snow flea anti-freeze protein (PP II, 2pne). Secondary structure class and PDB entry used to compute dihedral angles in parentheses.

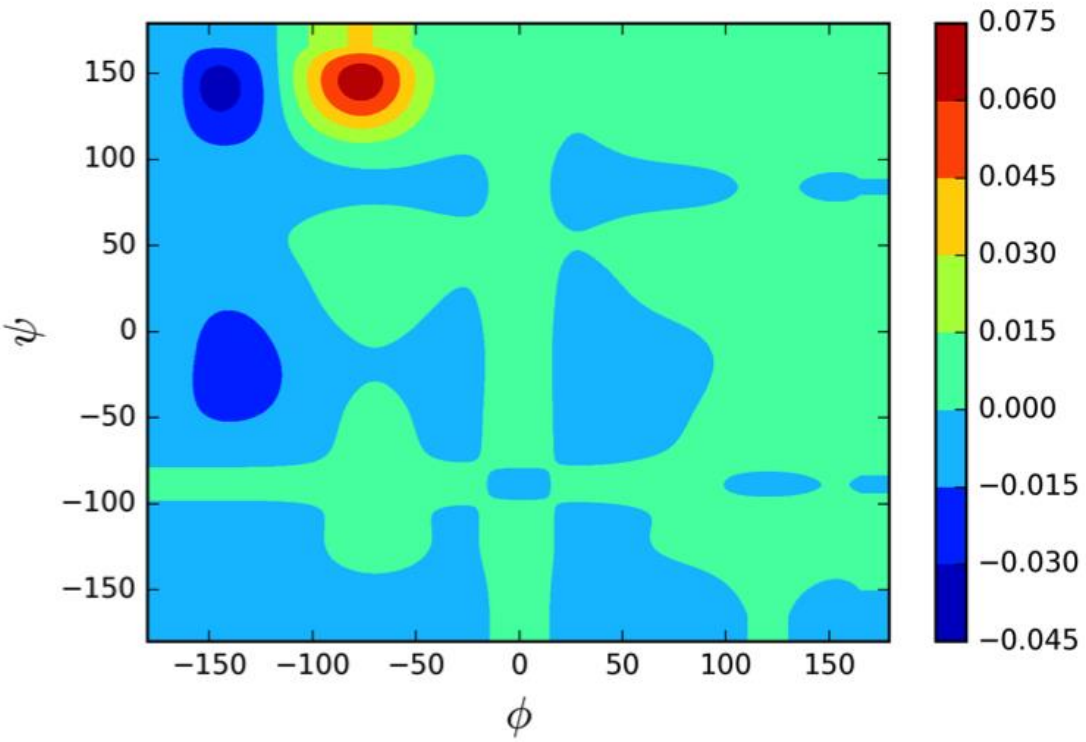

Figure S2. Ramachandran plot indicating the difference in the distributions of backbone dihedral angles (in degrees) populated throughout the MD simulations. The positive values correspond to regions more populated by elastase and the negative values correspond to regions more populated by concanavalin A (elastase - concanavalin A).
